# Soluble SARS-CoV-2 Spike glycoprotein: considering some potential pathogenic effects

**DOI:** 10.3389/fimmu.2025.1616106

**Published:** 2025-06-04

**Authors:** Bruno Azzarone, Nadine Landolina, Francesca Romana Mariotti, Lorenzo Moretta, Enrico Maggi

**Affiliations:** Tumor Immunology Unit, Bambino Gesù Children’s Hospital, IRCCS, Rome, Italy

**Keywords:** COVID-19, innate response, PASC, post-mRNA vaccine effects, SARS-CoV-2 infection, Spike protein

## Abstract

The soluble S1 subunit of Spike protein (SP) from the SARS‐CoV-2 of different variants of concern (VOCs) may directly bind and activate human NK cells *in vitro* through the engagement of the toll-like receptor (TLR) 2 and TLR4. This mechanism revealed a novel pathogenic role played by NK cells not only in the different phases of disease but also in the post-acute sequelae of COVID-19 (PASC) and some post-vaccination side effects. In addition to its binding to angiotensin-converting enzyme 2 (ACE2), which mediates virus attachment and cell entry, soluble SP triggers several active receptors/molecules expressed by many cells, inducing, in turn, type I/III interferon decrease, altered autophagy and apoptosis, the release of inflammatory cytokines and chemokines, complement activation and endothelial damage, which favour clotting events. In this review, we discuss the hypothesis that circulating SP, exerting multiple biological activities, can explain the heterogeneity of the clinical outcomes of severe COVID-19, PASC and post-vaccine-related effects. Recent reports have clearly indicated that soluble SARS-CoV-2 and post-vaccination SP trigger the same cascade of events, acting on the immune response and promoting defined adverse events. Factors hindering the pathological activity of soluble SP are the SP plasma levels, the age of the infected/vaccinated people and the efficiency of protein synthesis of ectopic targets triggered by soluble SP, as well as the specificity, the titre and the affinity of anti-SP antibodies elicited by the infection. At present, the risk/benefit ratio is largely in favour of vaccination; however, the excessive and persistent ectopic production of synthetic SP should be systematically analysed. This would allow for the identification of subjects at risk for major adverse events and to answer the urgent need for efficient vaccines that provide long-lasting activity with minimal side effects.

## Introduction

The coronavirus disease 2019 (COVID-19), caused by SARS-CoV-2, has been characterized by a rapid spread of infection, associated with overwhelming morbidity and mortality. Indeed, by January 2023, more than 671 million cases had been confirmed worldwide (260 million in Europe), with more than 6.71 million individuals succumbing to the disease (more than 2 million in Europe) (1). In surviving patients, SARS-CoV-2 may also cause post-COVID long-term symptoms due to organ or tissue damage. This condition, known as post-acute sequelae of COVID-19 (PASC), or long COVID-19 (2), affects approximately 10%–30% of non-hospitalized and 50%–70% of hospitalized patients. Importantly, 10%–12% of vaccinated patients also displayed PASC (3).

SARS‐CoV-2 is an enveloped virus, classified as a β2-coronavirus. Its single‐stranded RNA genome encodes seven structural viral proteins. Among them, the Spike Glycoprotein (SP) is expressed as a projection, approximately 20 nm long, at the virus surface. Three joined SP glycoproteins make up trimers that form structures resembling a crown surrounding the virion (4). The SP glycoprotein determines the specificity of the virus binding to angiotensin-converting enzyme 2 (ACE2), which also mediates virus cell entry. ACE2 is highly expressed on different cell types, including epithelia, endothelia, monocytes, phagocytes, dendritic cells (DCs) and type II pneumocytes (5).

## Pathologic effects induced by Spike glycoprotein

In ACE2^+^ cells and tissues, SP/ACE2 interaction is responsible not only for the entry of the virus but also for the decreased production of type I/III interferons that, in turn, may induce increased autophagy and apoptosis, a broad spectrum of pro-inflammatory signals with the release of cytokines and chemokines, increased TRL signalling, complement activation and endothelial damages favouring clotting events (6, 7).

SP can also induce cell activation and tissue damage through the direct engagement of cellular receptors/co-receptors and non-active molecules (such as TMPRSS2, CD26, CD147, CD209/CD209L TLR2/TLR7‐8, mannose-binding lectins, P53 and neuropilin-1) (6, 8–14), expressed on different cell types. This may result in the activation of different inflammatory pathways, leading to several endotypes and, in turn, disease phenotypes. In addition, it has been demonstrated that the S1 subunit of SP induces HLA‐E expression on epithelial cells, mediated by GATA3 activation, which interacts with NKG2A inhibitory receptors, negatively affecting NK cell function. The S1 subunit also displays epitopes with super-antigenic activity and/or that cross‐react with self‐antigens, favouring a non-specific polyclonal activation/exhaustion of T cells or contributing to the onset of complex autoimmune responses (6, 7, 15). In conclusion, SP may induce several pathological effects (recently named as “Spikeopathy”) through different mechanisms that lead to over-inflammation characterized by cytokine storm, thrombogenesis and endotheliitis-related tissue damage (7).

## SARS-CoV-2 and innate immune system dysregulation

SARS-CoV-2 infection may be accompanied by immune dysregulation and the release of cytokines, leading to a “cytokine storm”, mostly mediated by cells of the innate immunity. In these cells, the interaction of viral antigens with toll-like receptors (TLRs) has been proposed as the most relevant mechanism mediating COVID-19 pathogenesis (16).

Monocytes and macrophages are both infected by the virus (17). While macrophages are infected via ACE2, monocytes, which poorly express ACE2, are infected *via* CD147 (18). Among different monocyte subsets, the non-classical monocytes nMo1 are mostly activated by the interaction of CD147 with the S1 subunit. Notably, the proportion of circulating nMo1 is higher in severe than in mild disease and positively correlates with some inflammatory markers such as LDH and D-dimers. nMo1 numbers are also tightly associated with altered coagulation and inflammation parameters (19).

Another study showed that the proportion of non-classical (CD14^Low^CD16^+^) monocytes is significantly elevated in PASC patients at up to 15 months post-acute infection as compared to recovered non-PASC patients. Notably, in the PASC patients of this study, a significantly higher number of nMo1 contained the S1 subunit (20). Finally, cytokines as interleukin 12 (IL-12), IL-15 and IL-21, all relevant for NK cell proliferation and function, were not detected in some cases, suggesting that the monocytic/DC compartment may also be compromised (21). In this context, it has been recently shown that interactions, mediated by soluble factors and direct cell–cell contacts, between the NK cell subset CD56^bright^ CD16^−^ and monocytes, contribute to NK cell activation and dysfunction in severe COVID-19 patients through the production of elevated levels of pro-inflammatory cytokines, chemokines and TGF-β (22).

Neutrophils and neutrophil extracellular traps (NETs) have been implicated in the pathogenesis of COVID‐19. Indeed, activated neutrophils under increased PADI4 gene expression release DNA coated with histones, neutrophil elastase (NE), myeloperoxidase (MPO), cathepsin G and other proteins that form web-like structures such as NETs, which are able to trap and kill microorganisms due to the delivery of anti‐microbial molecules. However, NET release can cause tissue damage and can be highly detrimental to the host, considering histones’ cytotoxic effects on airway epithelia and vessel endothelia. Indeed, this may cause the inappropriate activation of both coagulation and thrombosis, thus heavily contributing to the endotheliopathy observed in severe COVID‐19 disease (23).

DC interaction with soluble SARS-CoV-2 S1 protein has been reported to promote the activation of key signalling pathways involved in inflammation, including MAPK, AKT, STAT1 and NF-κB, resulting in the production/release of distinct pro-inflammatory cytokines (24). Moreover, virus-like particles (VLPs) containing the receptor-binding motif of SARS-CoV-2 S1, upon interaction with DCs, promote the activation of the NF-κB pathway, the main pathway responsible for the synthesis of pro-inflammatory cytokines (25). This complex signalling network is likely activated through S1 binding to TLRs (primarily TLR2 and/or TLR4) expressed by DCs (10, 24, 25).

It has been suggested that both the tissue distribution and effector function of NK cells could be affected by SARS-CoV-2 infection. Remarkably, a prompt and strong NK cell response could determine a favourable outcome for infected patients (26). In this context, the infection modulates the phenotype of NK cells in COVID-19 patients by upregulating the expression of certain molecules, in particular of CD69, a surface marker linked to NK cell activation (27). However, several reports indicate that SARS-CoV-2 S1 may also induce NK cell dysfunction. For instance, NK cell function in the lung was impaired by S1 *via* the HLA-E/NKG2A interaction. In fact, S1 protein can induce HLA‐E expression on lung epithelial cells, which may interact with NKG2A and negatively affect NK cells. Another report reveals a more complex interaction showing that virus peptide/HLA-E induces the expansion of highly efficient NKG2C^+^ adaptive NK cells. An analysis of a cohort of COVID-19 patients in the acute phase of infection revealed that adaptive NK cells are induced irrespective of the Human cytomegalovirus (HCMV) infection. Overall, these data strongly suggest that NK cell responses to SARS-CoV-2 are mainly influenced by the balance between canonical and adaptive NK cells *via* the HLA-E/NKG2A/2C axis (28) and that NK cell dysfunction can heavily contribute to the immunopathogenesis of SARS-CoV-2 infection (29). Moreover, studies in Long-COVID patients have shown that while the adaptive CD56^+^CD57^+^NKG2C^+^ NK cell subset may be expanded, their anti-viral function is impaired (30). Another report underlines the occurrence of reduced NK cell numbers and compromised cytolytic activity in COVID-19 patients compared to healthy controls (21).

Of note, an expansion of an unusual NK cell subset exhibiting an altered phenotype (CD151^bright^CD9^+^) and impaired function has been observed in severe COVID-19 patients (31).

The divergent effects of the SP protein on NK cells could be partially explained by a recent paper (32) showing that, in short-term experiments, soluble SP from the Wuhan strain and other variants of concern (VOCs), as well as their S1 subunits, directly bind and activate purified NK cells, as demonstrated by enhanced activation marker expression, cytokine release and cytolytic activity induced primarily in the CD56^bright^ NK cell subset. Since ACE2 is neither expressed by NK cells nor induced by SP, different VOC-SPs can directly and efficiently bind both TLR2 and TLR4 and induce robust NK cell activation. In addition, VOC-SPs upregulate CD56^dim^ NK cell functions in recovered, but not in non-infected, individuals, suggesting the expansion of “adaptive” NK cells (32). This suggests that, at the disease onset, NK cells activated by SP may fight SARS-CoV-2 infection, while upon prolonged non-specific activation, in patients with severe disease and PASC, the engagement with SP can be detrimental, favouring the development of hypo-responsive NK cells (27). In this context, several papers have shown that the long-lasting stimulation of NK cells (both *in vitro* and *in vivo* with different stimuli) may cause NK cell hypo-responsiveness, as discussed in detail in a recent report (33).

Overall, the remarkable heterogeneity in COVID-19 outcomes may be due to variable degree of impairment of innate immune mechanisms that are frequently mediated by the SP protein. In patients with severe SARS-CoV-2 disease, heavily impaired innate immunity may cause the so-called “cytokine storm”, which is responsible for the excessive inflammatory response leading to acute respiratory distress syndrome (ARDS) and potentially to death (34).

## Circulating Spike protein during SARS-CoV-2 infection

SP, present in multiple copies in one virion particle and assembled into homotrimers, mediates viral entry by attaching to and fusing with the host cell membrane (35). It is cleaved into the S1 and S2 subunits by convertases, as transmembrane serine protease type II (TMPRSS2), cathepsins, furin or metalloproteases, which are expressed by infected cells (36). The S1 subunit binds the receptor ACE2 through the receptor-binding domain (RBD), while the S2 subunit links the S protein to the membrane, allowing viral entry (37). It has been calculated that, upon furin cleavage, approximately 50–100 soluble SP/viral particles are spread into the medium and bloodstream (38).

In mild COVID-19 patients, persisting active viral reservoirs have been found to release circulating SP protein. In addition, in severe COVID-19 patients with ARDS, the damage to endothelial cells and vascular leakage (39) can lead to the discharge of viral proteins such as SP and its S1 subunit into the blood. Thus, circulating SP can reach and further compromise different organs, acting substantially as a circulating toxin (40).

In this context, a recent study reported that SARS-CoV-2 S1 antigen was detectable in two-thirds of COVID-19 patients, revealing a significant correlation between high plasma S1 concentration and disease progression (40).

S1 can also be active when bound and expressed by exosomes. It has been demonstrated that exosomes in mild or severe COVID-19 patients contained SARS-CoV-2 Spike-derived peptides (41). Of note, 16 proteins were associated with exosomes in mild COVID-19 and were involved in pathways related to antigen processing and the presentation of exogenous peptides. In contrast, six proteins were detected in exosomes in severe COVID-19. These included complement factors, coagulation proteins, inflammation modulators and regulators of IL-6-mediated pro-inflammatory signalling (41).

In the blood, SP can activate platelets, which release coagulation factors, secrete inflammatory molecules and form leukocyte–platelet aggregates. Moreover, SP has been shown to bind fibrinogen, thus favouring blood clots (42). Finally, soluble SP can stimulate cardiac pericytes, which produce pro-inflammatory cytokines and promote microvascular disease (43).

SP can also activate all three complement system pathways, as shown by recent data showing that patients with detectable circulating SP protein had significantly higher levels of anaphylatoxins C3a/C5a than controls, which are correlated with a thrombophilic state (44).

**Figure 1:**
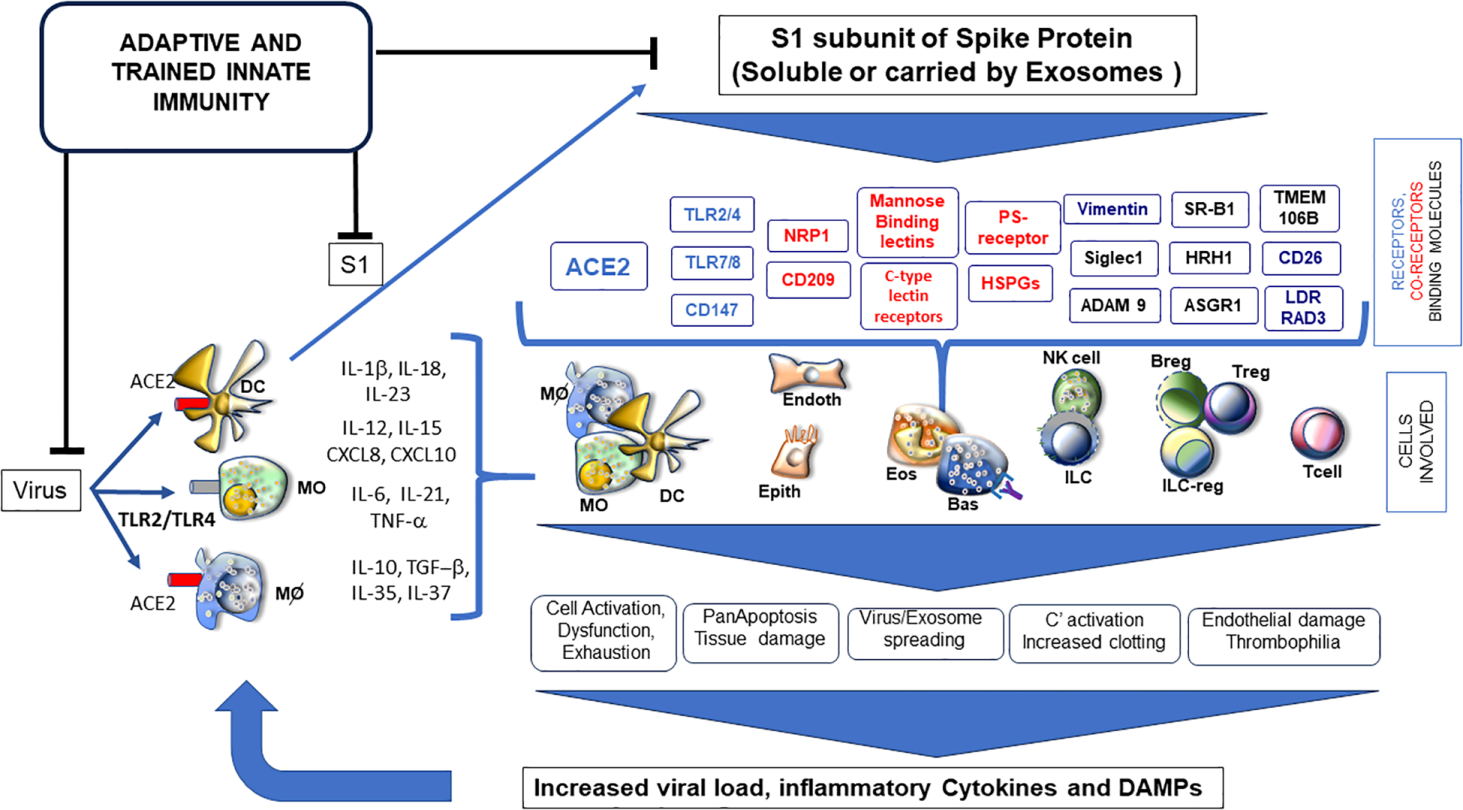
Soluble S1 subunit triggering a variety of receptors (blue) and co-receptors (red) and binding many surface molecules (black); it is responsible for activation/dysfunction of several cell types, highly influencing phenotypes of SARS-CoV-2 infection, PASC or post-vaccination events. ADAM9 (metalloproteinases as MMP9), ASGR1 (asialoglycoprotein receptor 1), CD147 (Basigin), C-type lectins (DC-SIGN and L-SIGN), HRH1 (histamine receptor 1), HSPGs (heparan sulfate proteoglycans), LDLRAD3 (low-density lipoprotein receptor class A domain containing), NRP1 (neuropilin 1), PS receptor (phosphatidylserine receptor), SIGLEC1 (sialoadhesin 1), SR-B1 (scavenger receptor class B type 1), TLR (toll-like receptors), TMEM106B (transmembrane protein 106B) and Vimentin.

Overall, the above-mentioned data could suggest that the SP itself, in particular the S1 subunit, as either a viral component or circulating forms (soluble or vehiculated by exosomes), may contribute to exert a pathogenic effect by inducing inflammation in different cell types and tissues in severe COVID-19 patients (**Figure 1**).

## Circulating Spike protein in long COVID-19 patients

COVID-19 patients recovered from the infection can experience long-term symptoms as a result of organ or tissue damage due to the virus. This condition is referred to as PASC or long COVID-19 (45, 46).

In this context, it has been reported that the presence of circulating SP in PASC patients persists up to 12 months after infection. This observation could suggest exploiting circulating SP as a useful bio-marker for PASC (47). Another report describes the persistent circulation of soluble and extracellular vesicle-linked SP in subjects with PASC even over 1 year after recovery from acute SARS-CoV-2 infection. Moreover, 30% of these patients tested positive for both SP and viral RNA, while none of the individuals without PASC were found to be positive, suggesting a contribution of SP to syndrome development (48). Other authors reported persisting circulating S1 subunit in 64% of post-infection patients with increased levels in individuals with ongoing PASC (49). Similar results were recently described in a proportion of PASC patients, suggesting that PASC with high circulating levels of the S1 subunit could represent a different cluster of patients (47).

A recent study quantified and mapped the SARS-CoV-2 organ tropism, showing long-term signs of infection in the brain, as well as across the body, even within non-respiratory sites (50). It has been suggested that soluble SP could also spread *via* the meninges into the brain, causing inflammation and, possibly, cell death (51). Notably, prolonged detectable levels of the circulating S1 subunit have been observed in children with multisystem inflammatory syndrome (MIS-C).

On the whole, several reports have indicated that circulating SP (both soluble and carried by exosomes) may be detectable in PASC patients’ sera, suggesting its possible involvement in the pathogenesis of the disease.

## Circulating Spike protein in vaccinated individuals

Natural mRNA is highly unstable, and for this reason, the synthetic mRNA coding for SP in COVID-19 vaccines is usually stabilized by the replacement of uridine with N1-methylpseudouridine (52). The mRNA is then packaged into lipid nanoparticles (LNPs) and injected into the deltoid muscle as a vaccine.

**Figure 2:**
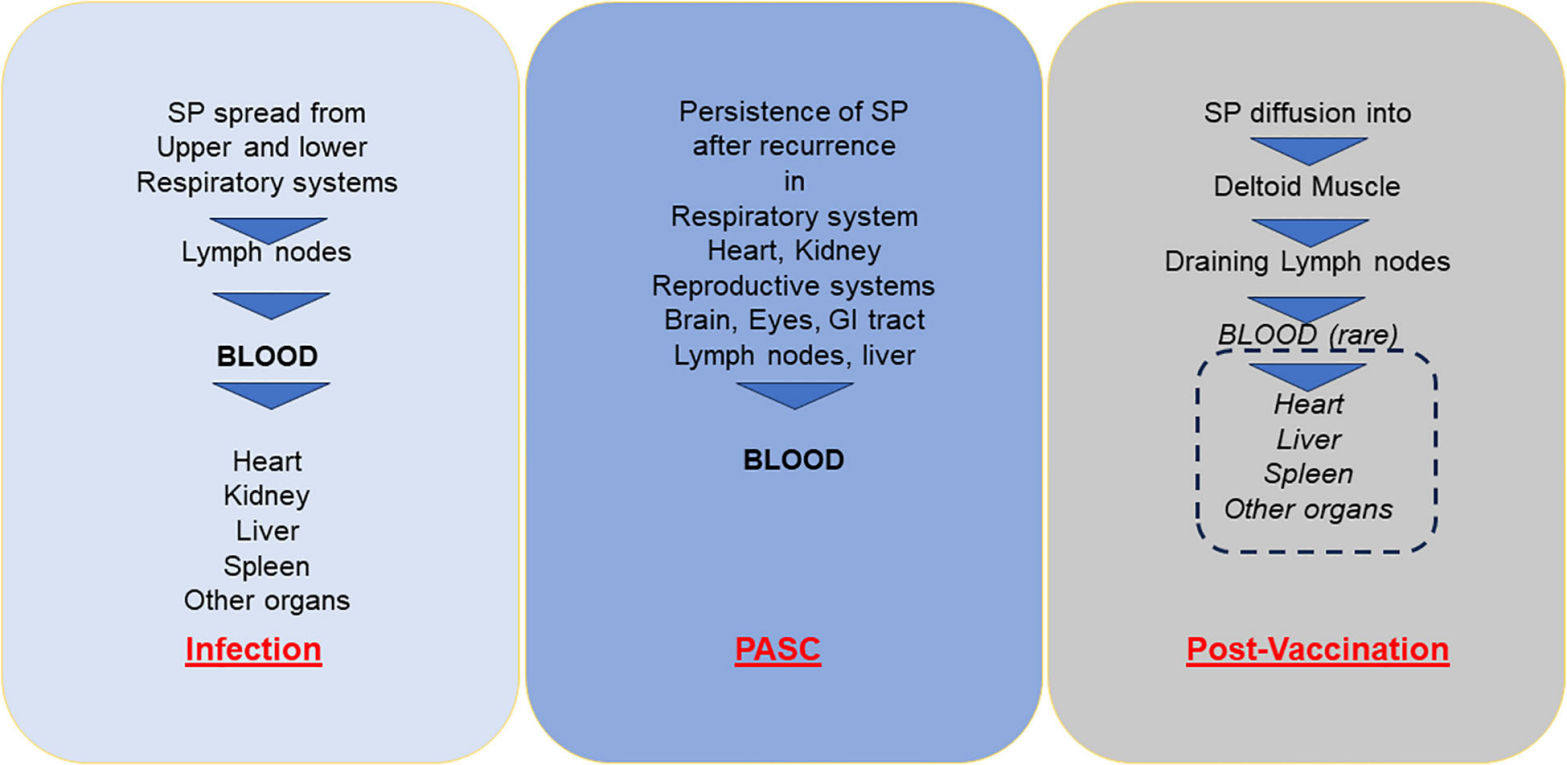
Diffusion of soluble (or exosome-bound) SP into bloodstream and different organs with acute infection in PASC and post-vaccination adverse events. SP, Spike protein; PASC, post-acute sequelae of COVID-19.

During the early phase of vaccine commercialization, the manufacturing companies believed that the persistence of mRNA-related SP production would be short and localised in the injected muscle. By contrast, more recent data showed that intramuscular injection causes an initial accumulation of LNPs at the injection site from where they migrate to proximal lymph nodes by passive draining or are actively transported by professional antigen-presenting cells (APC) and neutrophils. The remaining unprocessed vaccine particles may reach systemic circulation and, depending on the composition of the lipid shell, may reach the liver, spleen and other organs (7, 53, 54) (**Figure 2**).

In this regard, a recent paper reports that NK cell activation by the BNT162b2 vaccine may contribute to vaccine-induced inflammatory symptoms, reducing the timing of vaccine-induced antibody responses (55).

Thus, one could argue that this synthetic mRNA vaccine is excessively stable over a prolonged period (56), being able to generate pathogenic effects superposable to those induced by the viral SP during natural infection.

In agreement, a recent paper states that the same cascade of events triggered by SARS-CoV-2 infection was triggered by vaccine antigens. This possible suboptimal efficacy may account for preventing the infection (57). It is clearly demonstrated that the persistent SP after vaccination may contribute, together with the high viral load and the higher affinity of SP for its receptors, to the partial vaccine efficacy.

Indeed, the presence of vaccine mRNA and SP post-vaccination suggests that in its lipid-encapsulated form, mRNA may retain the ability to induce SP and its secretion in different susceptible cells (53, 58–61). Thus, circulating synthetic SP may penetrate various organs, including, in pregnant women, the placenta, with potential pathological consequences [for review, see (62)]. The excessive production of synthetic SP associated with post-vaccination adverse side effects has been reported: i) in vaccine-related thrombocytopenia occurring 10 days after vaccination; ii) in dermal keratinocytes and endothelial cells more than 3 months after vaccination associated with severe skin lesions, caused by varicella-zoster virus reactivation; and iii) in the right deltoid and quadriceps associated with diffuse myositis 1 month after injection of the mRNA vaccine into the left deltoid muscle (63).

**Figure 3:**
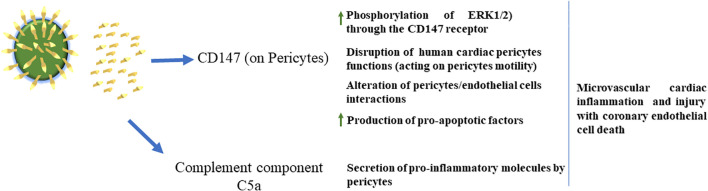
*Left*: SARS-CoV-2 can shed S proteins into the blood. *Middle:* S protein in the blood can activate CD147 receptors on pericytes to increase pericyte motility, activate pericyte ERK 1/2, cause impaired pericyte/endothelial cell interactions and cause pericytes to release pro-apoptotic factors. Pericytes also release pro-inflammatory factors via C5a. *Right:* S protein actions on pericytes cause damage to the brain and cardiovascular system, while detrimental effects on the eye, skeletal muscle and kidney remain unknown.

Along this line, a recent report has shown high levels of full-length unbound SP, detectable for up to 3 weeks after vaccination and eluded antibody recognition in adolescents who developed post-vaccine myocarditis, but not in the asymptomatic cohort (64). In this context, the *in vitro* exposure of primary human cardiac pericytes (PCs) to the SARS-CoV-2 recombinant SP of VOCs induced the phosphorylation/activation of the extracellular signal-regulated kinase 1/2 (ERK1/2), triggering the CD147 receptor. This may cause the secretion of pro-inflammatory molecules and the production of pro-apoptotic factors, leading to endothelial cell death. These data indicate that the soluble SP may induce PC dysfunction, thus contributing to endothelial injury (33) (**Figure 3**). These initial observations were subsequently confirmed by other studies (65–68).

Of note, in a Japanese population, the ratios among patients with myocarditis/pericarditis associated with SARS-CoV-2 mRNA vaccination vs. all vaccine-related adverse events were significantly higher than those in other populations. Indeed, among the registered adverse events, the percentages of myocarditis/pericarditis in Japan were found to be between 8%–11% and 5%–11%, with two types of mRNA vaccines (69), while, according to the European Medicines Agency (EMA), an adverse event spontaneous reporting database, in the whole vaccinated population, this percentage ranged between 0.55%–0.91% and 1.53%–3.03%, respectively (70). In general, these diseases were more frequent in people aged ≤30 years and men, associated with higher expression of IgG anti-myocardial cell antibodies; this suggests that mRNA vaccines increase IgG expression levels and, in turn, the risk of myocarditis in young compared to older people. Importantly, synthetic SP has been identified in endomyocardial biopsies of patients with myocarditis up to nearly 2 months after COVID-19 vaccination, supporting the link between the accumulation of SP in the cardiac tissue and its damage (71).

## Factors interfering with Spike biological activity

There is a general consensus that the induction of adverse events by soluble SP depends on its plasma levels. Indeed, high levels (in the range of ng/mL) are required in mice to mimic the endotheliopathy observed in severe COVID-19 case series (72). Such levels have been essentially observed in severe COVID-19 patients, while higher SP levels correlated with intensive care unit (ICU) admission (40). By contrast, in people vaccinated with mRNA vaccine, soluble SP can be detected 1 day after the first injection, with plasma levels up to 150 pg/mL (40). Such levels are likely too low to trigger permanent endothelial cell damage, as shown by a recent report, which demonstrates that circulating SP in this range only caused a limited and transient endothelial damage, which reversed to normal levels 2 days after vaccination (73).

It has recently been proposed that excessive and persistent ectopic production of the synthetic SP may depend on “factors specific to the recipient organism, such as more efficient protein synthesis, especially in young people, or localization of COVID-19 vaccine RNA-containing nanoliposomes in tissues or organs with intrinsically high protein synthesis capacity (e.g, liver, ileum, heart, skeletal muscle)” (74). In addition, since exosomes carrying SP can be detected in the plasma of vaccinated subjects up to 4 months after mRNA vaccine (53, 75, 76), it is important to establish whether these exosomes mediate a favourable immune response or rather an excessive inflammatory response (41). Indeed, the presence *per se* of SP-carrying exosomes is not sufficient to establish their pathological role, and it is necessary to determine their phenotype and the effector molecules conveyed (41).

Finally, an important factor interfering with SP activity is represented by the type and intensity of adaptive immune response towards the virus. The isotypes/subclasses, levels and quality (neutralizing, opsonizing, etc.) of anti-S1 Abs, elicited by infection or vaccination, can deeply condition all SP biological effects, modifying its interactions with different receptors or other protein targets. Notably, the anti-S1 response is also able to impair the activity of exosomes expressing SP. The immune status before infection could also be relevant; at present, correlative studies comparing (single or multiple) defects of humoral response and high SP plasma levels are missing, as well as lacking any direct evidence linking some immune defect with possible SP effects.

## Limitations and controversies

Several experiments show that SP affects *in vitro* the function of several cells of the innate immunity, and it is pathogenic for several types of cells (10, 17, 24, 25, 29, 32, 43). Nevertheless, the situation *in vivo* appears to be much more complex. In this context, the pathogenic role of free SP to determine post-vaccination tissue alterations and clinical side effects (as myocarditis), even if clearly confirmed by different studies (62–70), leaves some questions still open. For instance, the extremely low incidence of the pathologies due to mRNA COVID-19 vaccines in the face of billions of doses overall administered in the last 3 years has not been convincingly explained. In addition, the free SP levels are variable and usually very low in vaccinated people in comparison with those present in the plasma of severe COVID-19 patients. Other unknown mechanisms, including the status of the immune system, the effectiveness of anti-SARS-CoV-2 immunity induced by vaccination and comorbidities, or genetic factors (69), must be taken into consideration. At present, controlled studies on very large cohorts of patients (as correlative studies among SP levels or inducible anti-SP response or their persistence, with vaccination side effects) are still missing and have become mandatory to understand whether soluble SP protein acts as a contributing cause or is the main cause of the post-vaccination adverse events.

## Conclusions

SARS-CoV-2 SP was initially considered the viral structure responsible for binding and entry into target cells, but essentially deprived of any pathogenic effects. However, several studies have now shown that SP, either as a structural component of the virus or as a circulating protein carried by exosomes, may exert harmful effects on different cells and tissues, potentially causing cellular damage that can even lead to organ failure in both severe disease and PASC. Soluble SP, neo-synthesized after vaccination with anti-SARS‐CoV-2 mRNA vaccines, has been associated with rare adverse side events. In these patients, the interaction of the SP with different protein targets of different types of cells (NK, dendritic, monocytic and epithelial cells) induces the changes in their cellular pathways, leading to the overrated release of different molecular effectors, shifts from classical to non-classical populations and the activation of neutrophils, which in turn trigger a cascade of events involved in defined adverse effects.

At present, the risk/benefit ratio is largely in favour of the vaccination; however, the above-mentioned studies have suggested that an age difference in the processing and clearance of SP translated from the mRNA vaccine may cause the excessive and persistent ectopic production of the synthetic SP. These events have not been systematically analysed in humans, and future research should clarify the fine mechanisms by which inappropriate localization and excessive SP synthesis can develop in certain (genetically defined) individuals.

Finally, since some issues limiting the full efficiency of the presently employed vaccines have recently been raised, there is an urgent need to develop new vaccines able to inhibit viral spread and provide anti-viral long-lasting immune-mediated responses (77).
